# Perception Toward Exposure Risk of COVID-19 Among Health Workers in Vietnam: Status and Correlated Factors

**DOI:** 10.3389/fpubh.2021.589317

**Published:** 2021-05-25

**Authors:** Xuan Thi Thanh Le, Quynh Thi Nguyen, Brenda Onyango, Quang Nhat Nguyen, Quan Thi Pham, Nhung Thi Kim Ta, Thao Thanh Nguyen, Huong Thi Le, Linh Gia Vu, Men Thi Hoang, Giang Thu Vu, Carl A. Latkins, Roger C. M. Ho, Cyrus S. H. Ho

**Affiliations:** ^1^School of Preventive Medicine and Public Health, Hanoi Medical University, Hanoi, Vietnam; ^2^Rollins School of Public Health, Emory University, Atlanta, GA, United States; ^3^Nuffield Department of Medicine, University of Oxford, Oxford, United Kingdom; ^4^Institute for Global Health Innovations, Duy Tan University, Da Nang, Vietnam; ^5^Faculty of Medicine, Duy Tan University, Da Nang, Vietnam; ^6^Center of Excellence in Evidence-Based Medicine, Nguyen Tat Thanh University, Ho Chi Minh City, Vietnam; ^7^Bloomberg School of Public Health, Johns Hopkins University, Baltimore, MD, United States; ^8^Department of Psychological Medicine, Yong Loo Lin School of Medicine, National University of Singapore, Singapore, Singapore; ^9^Institute for Health Innovation and Technology (iHealthtech), National University of Singapore, Singapore, Singapore; ^10^Department of Psychological Medicine, National University Health System, Singapore, Singapore

**Keywords:** hospital staff, Vietnam, perception, exposure risk, COVID-19

## Abstract

**Background:** Hospital staff are at the frontline for the prevention and control of COVID-19. Understanding their perception of exposure risk is, therefore, important during the early phase of this pandemic. In this study, we evaluated the perception regarding risk of exposure to COVID-19 among Vietnamese hospital staff in Vietnam.

**Method:** A cross-sectional online study was carried out to collect demographic data and risk exposure perception during the second week of the national lockdown in April 2020 in Vietnam. Seven hundred and forty two hospital staff were recruited using the snowball sampling to answer 5-point Likert scale questions regarding their risk exposure perception. Exploratory factor analysis (EFA) was used to examine the construct validity of the questionnaire. Pearson coefficient analysis and multivariable regression models were applied to identify factors associated with the perceived COVID-19 exposure risk.

**Results:** Participants perceived a high risk of being infected with SARS-CoV-2 (score = 3.4, SD = 0.8). They also perceived the workplace response to COVID-19 as inadequate (score = 2.0, SD = 0.5). In particular, participants who worked in the emergency or intensive care departments were more likely to perceive an exposure risk, compared to those in infectious disease control departments (Coef. = −0.38, 95%CI: −0.74; −0.02). Participants from central regions perceived a lower risk of exposure to COVID-19 than those from northern regions (OR = 0.52, 95%CI: 0.28–0.96). Nurses were less likely than doctors to report being at risk of exposure to COVID-19 (OR = 0.56, 95%CI: 0.33–0.95).

**Conclusions:** We identified a high level of perceived risk regarding COVID-19 exposure among hospital staff during the unprecedented lockdown period in Vietnam. A comprehensive approach, incorporating improved risk communications, safety training and psychological support programs, for all hospital staff, including nurses and those residing in high population density areas, might further strengthen the national effort to control the pandemic.

## Introduction

Despite the recent progress in COVID-19 vaccine development and implementation, SARS-CoV-2, the virus which causes COVID-19, remains a threat to the global health care systems (1, 2). As of March 16, 2021, the virus has spread worldwide, resulting in 120,164,106 million confirmed cases and 2,660,422 death (3). Hospital staff, including primary care doctors, specialists, nurses, and administrative staff, are at the forefront of the screening, diagnosis, and treatment of patients with COVID-19. As a result, they experience a disproportionate risk of exposure to the virus. Such an exposure becomes more likely because of their closer, more frequent contact and longer working hours during the pandemic compared to their activities before the pandemic (4–6).

Previous studies have in fact revealed that health care workers perceived a high risk of exposure to COVID-19 (7, 8). Staff perceived they were more at risk than their family members during the COVID-19 pandemic to exposure of the virus. However, they were more worried for their family members than for themselves. In addition, health care workers reported that Covid-19 had a significant impact on their everyday workload (6).

This high perception of risk might be explained by differences in the pandemic response strategies and provision of resources to protect this critical workforce among countries. For example, countries vary in their capacity and policies for distributing adequate personal protective equipment (PPEs) to hospital staff. In Singapore, medical staff protection and surveillance strategy were ordered, including risk-based PPEs, surveillance for staff fever and sickness, and enhanced medical surveillance of unwell staff (9). In Vietnam, hospital staff must comply with clinical practice principles to protect themselves and to reduce infections (10). In 2020, the Vietnamese government ordered an unprecedented 14-day lockdown to prevent and control COVID-19 (11). In addition, testing services were provided for staff who exhibited COVID-19 symptoms, who had close contacts with COVID-19 patients, or who had worked where infections of health care workers were reported.

Indeed, having a high risk of exposure to COVID-19 might negatively impact the well-being and performance of health workers. Nevertheless, as of March 16, 2021, most studies about COVID-19 in Vietnam remain largely clinical, epidemiological, or vaccine-related. There exists a great gap in our understanding regarding the perceived risk of exposure to this disease among hospital staff (12, 13). In this study, we assessed the perception of COVID-19 exposure risk among Vietnamese hospital staff in Vietnam in 2020. Such a knowledge might further guide healthcare leaders and policymakers in the national effort to holistically support this important workforce, who plays an important role in the current pandemic control and rollout of vaccines in the near future.

## Methods

### Study Setting and Participants

A cross-sectional study was carried out during the second week of the national lockdown in April 2020 in Vietnam. During this period, it was highly recommended by the national government that all Vietnamese citizens stayed at home and remained socially distant in order to control the transmission of SARS-CoV-2. Participants who met the following inclusion criteria took part in the survey: (1) they self-reported working as a healthcare worker at health centers or hospitals; (2) agreed to take part in the research by signing the online informed consent document; (3) could access web-based questionnaire; (4) could read, understand, and respond to the questionnaire.

### Sample and Sampling

Respondents were recruited using the snowball sampling technique, which is based on having the participants refer subsequent participants whom they know. A core group of 15 participants at the Hanoi Medical University was established; these participants had a high chance of knowing others through their networks of medical students and staff in different medical universities in Vietnam. A link to the online questionnaire was provided to people in this core group. After finishing the questionnaire, they sent the link of questionnaire to their close contacts via Email, Facebook, or Zalo mobile applications. Study participants include health care workers in hospitals and health care centers along with medical university staff throughout 63 provinces of Vietnam. In total, 742 participants took part in this study.

### Measures and Instruments

An online questionnaire was designed on the Survey Monkey platform (https://www.surveymonkey.com/), which is a low-cost, less time-consuming, and universally accessible platform for collecting data nationwide. The measurements collected include socio-demographic characteristics and perceived risk of exposure to COVID-19.

#### Socio-Demographic Characteristics

We collected self-reported demographic characteristics of respondents, including region (northern, central, or south), sex (male or female), marital status (single/separated/widowed or married), living with (family/friends or alone), education level (university and below, or postgraduate degree), department (Emergency/Intensive Care, Internal Medicine, Surgery/Obstetrics/Pediatrics, Imaging Diagnosis/ Scientific Laboratory/Clinic, Administrative Offices, Infectious Disease/Infection Control, Preventive Medicine/Public Health/Nutrition, or Others), level of hospital (Central level, Provincial level, District health center, or Others), age, and years spent in their career.

#### Perceived Risk of Exposure to COVID-19

Respondents answered 15 questions about their perceptions regarding the risk of being exposed to COVID-19, risk of being infected with SARS-CoV-2, and the workplace response to COVID-19. Each question was rated by using a five-point Likert scale with 1 = “Strongly disagree,” 2 = “Disagree,” 3 = “Neutral sentiment,” 4 = “Agree,” and 5 = “Strongly agree.”

Respondents also self-reported the frequency of perceived exposure to COVID-19 (not exposed, every day, several times per week, seldom, or do not know), the number of diagnosed and undiagnosed COVID-19 patients to whom participants believed they were exposed, and their willingness to look for another job to avoid the risk of contracting the virus (Yes or No).

### Data Analysis

STATA 15.0 (StataCorp LP, College Station, TX) was used to analyze the data. We applied exploratory factor analysis (EFA) to assess the construct of the measurement, and Cronbach's alpha was used to measure the internal consistency of the questionnaire. In the factor analysis, the cut-off mean eigenvalue was above 0.5 to select the factor. The cut-off point for factor loading was defined at a value of 0.4. Three factors were grouped, which included: (1) Individuals at high risk of SARS-CoV-2 transmission, (2) Risk perception of being infected with SARS-CoV-2; (3) Perceived response of workplace to COVID-19 (14).

Descriptive statistics were used to examine the frequency, percent, mean, and standard deviation. Inferential statistics were applied to compare three subject groups by the *t*-test or Mann Whitney test for nominal variables and by the Fisher-exact test or Chi-square test for ordinal variables. Multivariable regression models and Pearson coefficient analysis were applied to identify factors associated with the risk of exposure to COVID-19. A set of candidate variables were identified, including: (1) participants' socio-demographic characteristics, (2) the number of diagnosed and undiagnosed COVID-19 patients to whom participants believed to be exposed. This set variable was first entered in the full regression models (Appendix 1). In order to obtain reduced models, stepwise forward selection strategies were utilized with a log-likelihood ratio test at a *p*-value of 0.20. Statistical significance was defined at a *p*-value of < 0.05.

### Ethical Consideration

The Review Committee at the Institute for Preventive Medicine and Public Health, Hanoi Medical University approved this study on March 27, 2020. The purpose of the research and informed consent forms were provided to participants online. Participation was voluntary, and the anonymity was assured. Respondents were informed of the options to decline to participate and withdraw from the online survey at any time.

## Results

Table 1 summarizes socio-demographic characteristics of the participants. More than half of the participants were female (65.8%). Most participants came from the northern region (71.6%), were married (78.7%), and had obtained university or lower degree(s) (61.1%). Almost all of the participants lived with their families or friends (91.9%). The average age was 36.3 years (SD = 9.1), and the average length of working experience was 11.4 years (SD = 8.8).

**Table 1:** Demographic characteristics of respondents.

	**Job type**								***P*-value**
	**Doctor**		**Nurse**		**Others**		**Total**		
	***n***	**%**	***n***	**%**	***n***	**%**	***n***	**%**	
Total	379	51.1	208	28.0	155	20.9	742	100.0	
**Region**									
Northern	269	71.0	159	76.4	103	66.5	531	71.6	0.20
Central	76	20.1	33	15.9	40	25.8	149	20.1	
South	34	9.0	16	7.7	12	7.7	62	8.4	
**Gender**									
Male	174	45.9	35	16.8	45	29.0	254	34.2	<0.01
Female	205	54.1	173	83.2	110	71.0	488	65.8	
**Marital status**									
Single/separated/widowed	73	19.3	40	19.2	45	29.0	158	21.3	0.03
Married	306	80.7	168	80.8	110	71.0	584	78.7	
**Living with**									
Family/friends	353	93.1	198	95.2	131	84.5	682	91.9	<0.01
Alone	26	6.9	10	4.8	24	15.5	60	8.1	
**Education level**									
University and below	157	41.4	186	89.4	110	71.0	453	61.1	<0.01
Postgraduate degree	222	58.6	22	10.6	45	29.0	289	38.9	
**Department**									
Emergency-intensive care	25	6.6	38	18.3	0	0.0	63	8.5	<0.01
Internal medicine	56	14.8	36	17.3	3	1.9	95	12.8	
Surgery-obstetrics-pediatrics	53	14.0	34	16.3	4	2.6	91	12.3	
Imaging diagnosis-scientific laboratory—clinic	64	16.9	24	11.5	31	20.0	119	16.0	
Administrative offices	48	12.7	12	5.8	50	32.3	110	14.8	
Infectious disease-infection control	24	6.3	13	6.3	5	3.2	42	5.7	
Preventive medicine—public health -Nutrition	41	10.8	14	6.7	31	20.0	86	11.6	
Others	68	17.9	37	17.8	31	20.0	136	18.3	
**Level of hospital**									
Central level	127	33.5	47	22.6	42	27.1	216	29.1	
Provincial level	96	25.3	90	43.3	46	29.7	232	31.3	
District health center	59	15.6	20	9.6	22	14.2	101	13.6	
Others	97	25.6	51	24.5	45	29.0	193	26.0	
	**Mean**	**SD**	**Mean**	**SD**	**Mean**	**SD**	**Mean**	**SD**	***P*****-value**
Age (year)	37.6	9.7	35.2	7.4	34.7	9.3	36.3	9.1	<0.01
Years spent in a career (years)	12.2	9.5	11.1	7.6	9.9	8.1	11.4	8.8	0.05

Table 2 shows the participants' perceived risk of exposure to COVID-19. There were 33.8% of participants who strongly agreed that their agency leaders would not provide the necessary medical services if they were infected with SARS-CoV-2, followed by a high number of participants expressing a disagreement to continue working at the current health facility, since it may be contaminated with SARS-CoV-2 (26.4%). It is worth noting that only 1% of participants strongly agreed that they should not take care of COVID-19 patients, and a similarly small number of participants believed their chances of survival were low if they were infected with the virus. Nevertheless, the score of 0.38 was reported for participants who “accept if a colleague quit because he/she was afraid of COVID-19.”

**Table 2:** Perceived risk of exposure to COVID-19.

**Exposure risk to COVID-19**	**Strongly agree**		**Perceived risk of COVID-19 transmission**	**Perceived risk of being infected with SARS-CoV-2**	**Perceived COVID-19 response of the workplace**
	***n***	**%**			
I am most concerned about transmitting SARS-CoV-2 to my family member	102	19.3	0.76		
I am most concerned about transmitting SARS-CoV-2 to my colleagues	61	11.5	0.87		
I am most concerned about transmitting SARS-CoV-2 to my close friends	59	11.2	0.87		
I am most concerned about transmitting SARS-CoV-2 to others	58	11.0	0.89		
Feel the job put me at high risk of being exposed to COVID-19	92	15.1		0.72	
People around me are worried about my health	46	8.7		0.56	
Fear of being infected with SARS-CoV-2	39	6.4		0.57	
My family believes that I am at high risk of COVID-19 exposure	33	6.2		0.78	
My job puts people near me at high risk of COVID-19 exposure	33	6.2		0.68	
People around me are worried they might get COVID-19 from me	24	4.5		0.61	
The agency leader will not provide me with the necessary medical services if I were infected with SARS-CoV-2	179	33.8			0.69
Disagree to continue working at a current health facility, since it may be contaminated with COVID-19	161	26.4			0.67
Accept if a colleague quit because they are afraid of COVID-19	10	1.9			0.38
Should not take care of COVID-19 patients	6	1.0			0.62
If infected with SARS-CoV-2, I believe my chance of survival is low	5	1.0			0.50
Cronbach's alpha			0.92	0.80	0.49
Mean			3.6	3.4	2.0
SD			0.8	0.6	0.5

Table 3 indicates that doctors self-reported they were at greater daily risk of exposure to COVID-19 compared to nurses (53.8 and 51.9%, respectively, *p* < 0.05). There were 13.5% of participants who did not know their exposure risk to COVID-19. Only 0.7% of all nurses reported looking for another job, whereas 2.1% of doctors and 3.1% people with other types of jobs reported looking for another job. Nonetheless, these differences among job types were not statistically significant (*p* > 0.05). On average, hospital staff reported being exposed to 1 (SD = 6.5) COVID-19 patient with a known diagnosis and 12.3 (SD = 83.6) patients with diagnosis that was only known afterwards. The scores of the perceived risk of exposure to COVID-19, being infected with SARS-CoV-2, and perceptions of the workplace response to COVID-19 were 3.6±0.8, 3.4±0.6, and 2.0±0.5, respectively.

**Table 3:** Perception regarding the risk of exposure to COVID-19.

	**Job type**								***P*-value**
	**Doctor**		**Nurse**		**Others**		**Total**		
	***n***	**%**	***n***	**%**	***n***	**%**	***n***	**%**	
**Risk of exposure to COVID-19**									
Are not exposed to risk factors	33	8.7	31	14.9	30	19.4	94	12.7	0.03
Everyday	204	53.8	108	51.9	69	44.5	381	51.4	
Several times per week	47	12.4	15	7.2	18	11.6	80	10.8	
Seldom	47	12.4	22	10.6	18	11.6	87	11.7	
Do not know	48	12.7	32	15.4	20	12.9	100	13.5	
Looking for another job to avoid the risk of COVID-19 exposure	7	2.1	1	0.7	4	3.1	12	2.0	0.30
**Information domain**	**Mean**	**SD**	**Mean**	**SD**	**Mean**	**SD**	**Mean**	**SD**	***P*****-value**
Number of diagnosed COVID-19 patients to whom you were exposed	0.9	6.8	0.9	4.3	1.3	7.6	1.0	6.5	0.59
Number of undiagnosed COVID-19 patients to whom you were exposed	10.0	74.7	14.6	103.0	16.6	85.2	12.3	83.6	0.67
Perceived risk of transmitting SARS-CoV-2 (score: 1 to 5)	3.6	0.8	3.7	0.9	3.4	0.9	3.6	0.8	0.02
I am most concerned about transmitting the virus to my family member	3.8	0.9	3.9	0.9	3.7	1.0	3.8	0.9	0.11
I am most concerned about transmitting the virus to my close friends	3.4	1.0	3.6	1.1	3.3	1.0	3.5	1.0	0.08
I am most concerned about transmitting the virus to my colleagues	3.7	0.9	3.8	0.9	3.4	0.9	3.6	0.9	<0.01
I am most concerned about transmitting the virus to others	3.6	0.9	3.7	1.0	3.4	1.0	3.6	0.9	0.04
Perceived risk of being infected with SARS-CoV-2 (score: 1 to 5)	3.5	0.6	3.4	0.7	3.2	0.6	3.4	0.6	<0.01
Feel that the job put me at high risk of being exposed to COVID-19	3.7	0.9	3.7	1.0	3.3	0.9	3.6	0.9	<0.01
Fear of being infected with the virus	3.3	0.9	3.4	1.0	3.0	0.9	3.3	0.9	<0.01
My family believes that I am at high risk for COVID-19	3.5	0.9	3.2	1.0	3.2	0.9	3.4	0.9	<0.01
My job puts people near me at high risk of COVID-19 exposure	3.5	0.8	3.3	0.9	3.2	0.9	3.4	0.9	<0.01
People around me are worried about my health	3.6	0.8	3.8	0.8	3.4	0.9	3.6	0.8	<0.01
People around me are worried that they might get COVID-19 from me	3.2	0.9	3.3	0.9	3.0	0.9	3.2	0.9	0.02
Perceived response of the workplace to COVID-19 (score: 1 to 5)	2.0	0.5	2.0	0.5	2.1	0.5	2.0	0.5	0.01
Should not take care of COVID-19 patients	2.0	0.9	1.8	0.8	2.0	0.8	1.9	0.8	0.11
Disagree to continue working at a current health facility, since it may be contaminated with COVID-19	1.9	0.9	2.0	1.0	2.0	0.8	2.0	0.9	0.45
Accept if a colleague quit because they are afraid of COVID-19	2.5	1.0	2.4	0.8	2.6	0.9	2.5	0.9	0.34
The agency leader would not provide me with the necessary medical services if I were infected with the virus	1.8	0.8	1.8	0.9	2.1	0.9	1.9	0.9	<0.01
If infected with the virus, I believe my chances of survival are low	1.9	0.7	1.8	0.8	1.9	0.7	1.9	0.7	0.33

Table 4 shows participants' characteristics associated with having a greater perception of risk of exposure to COVID-19 and perceived risk of transmitting the virus to others. Participants who came from the central region had less perceived risk of exposure to COVID-19 than people from the north region (OR = 0.52). Female participants were more likely to perceive a greater risk of exposure to COVID-19 (OR = 2.23) compared to their male counterparts. Participants who were married had better perception of the risk of transmitting SARS-CoV-2 (Coef. = 0.28; 95% CI = 0.06–0.51) and more positive perceptions about the workplace response to COVID-19 (negative perception: Coef. = −0.19; 95% CI = −0.31– −0.07) compared to others.

**Table 4:** Associated factors of exposure risk to COVID-19.

**Demographic**	**Risk of exposure to COVID-19**		**Perceived risk of transmitting SARS-CoV-2**		**Perceived risk of being infected with SARS-CoV-2**		**Perceived response of the workplace to COVID-19**	
	**OR**	**95% CI**	**Coef**.	**95% CI**	**Coef**.	**95% CI**	**Coef**.	**95% CI**
**Region (vs. Northern)**								
Central	0.52^**^	0.28; 0.96			−0.13	−0.30; 0.03		
**Gender (vs. male)**								
Female	2.23^***^	1.40; 3.55						
**Living with (vs. family/friends)**								
Alone					0.24^*^	−0.01; 0.49		
**Marital status (vs. single/separated/widowed)**								
Marriage			0.28^**^	0.06; 0.51			−0.19^***^	−0.31; −0.07
**Job types (vs. doctor)**								
Nurse	0.56^**^	0.33; 0.95	0.18^*^	−0.02; 0.37			−0.15^**^	−0.27; −0.03
Others					−0.11	−0.27; 0.05	0.12^*^	−0.00; 0.24
**Level of hospital (vs. central level)**								
Others							0.12^**^	0.02; 0.23
**Department (vs. emergency-intensive care)**								
Internal medicine			0.16	−0.08; 0.40	0.13	−0.07; 0.33		
Imaging diagnosis-scientific laboratory—clinic	0.45^***^	0.25; 0.82						
Administrative offices			−0.20^*^	−0.44; 0.04	−0.26^***^	−0.46; −0.06		
Infectious disease-infection control			−0.38^**^	−0.74; −0.02	−0.26^*^	−0.54; 0.03		
Preventive medicine-public health-nutrition			−0.28^**^	−0.53; −0.04	−0.23^**^	−0.43; −0.03		
Others	0.66	0.36; 1.21			−0.18^**^	−0.35; −0.00	0.13^**^	0.00; 0.25
Length of work experience (unit: years)	0.98^*^	0.95; 1.00	−0.01^***^	−0.02; −0.00	0.01^*^	−0.00; 0.01		
Number of diagnosed COVID-19 patients to whom you believe to be exposed					0.01^*^	−0.00; 0.02		
Number of undiagnosed COVID-19 patients to whom you believe to be exposed	0.98^*^	0.95; 1.00						

*** *p < 0.01*,

** *p < 0.05*,

* *p < 0.1*.

We also observed that participants who worked in the Imaging Diagnosis-Scientific Laboratory were less likely to be perceptive of the risk of exposure to COVID-19, compared to those who worked in the Emergency-Intensive Care departments (OR = 0.45). Participants who worked in the Emergency-Intensive Care department had higher scores on the perceived risk of transmitting SARS-CoV-2 than those who worked in the Infectious Disease-Infection Control departments (Coef. = −0.38, 95%CI: −0.74; −0.02) and in the Preventive Medicine-Public Health-Nutrition departments (Coef. = −0.28,95%CI: −0.53; −0.04). Lastly, participants who were nurses had lower perceived exposure risk (OR = 0.56) and negative perceptions about the workplace response to COVID-19 (Coef. = −0.15) compared to participants who were doctors.

## Discussion

Hospital staff play a critical role in the COVID-19 pandemic control and response. They are, however, also at a higher risk of SARS-CoV-2 infections and can be a source of viral transmission in the community (10). In addition to ensuring proper infection protection for hospital staff, it is, therefore, also important to assess in order to improve their perception regarding the risk of exposure to the disease. The present study assessed such a perceived risk by using a cross-sectional online survey to evaluate the exposure risk perception during the unprecedented Vietnamese lockdown. Our findings provide useful insights into further developing a risk-based pandemic intervention to support hospital staff.

In this study, participants from the central region were less likely to report exposure to COVID-19 than those from the northern region (OR = 0.52), 95%CI: 0.28–0.96). Population differences may explain this finding. The Northern region has ~1,060 people/km^2^ (15). Moreover, hospitals in this region often experience over-capacity. This finding is consistent with another study in Vietnam, which links the differences in geographical differences to exposures (12). Regarding the perceived exposure risk, nurses reported a lower daily risk of exposure than doctors (51.9 and 53.8%, respectively). In addition, nurses were more likely to report their exposures to COVID-19 than doctors (OR = 0.56), 95%CI: 0.33–0.95). These findings may be due to the nature of the work in which doctors often perform procedures in closer contact with the patient than nurses, albeit at a lower patient-contact frequency, such as intubation. Therefore, a pandemic support intervention for health care workers should be prioritized for population-high density areas and medical doctors. Such an intervention should include design software to follow daily exposure risk.

We observed, in this study, a high level of perception regarding the risk of being exposed to COVID-19 (score = 3.4). This finding is consistent with a previous study conducted in Ho Chi Minh City in 2020, which revealed that Vietnamese healthcare workers had a higher than mid-point level of exposure risk perception (score = 7.65) (12). Other studies also suggest healthcare workers appear to have relatively high exposure risk perceptions regarding COVID-19 (7, 8, 16). Furthermore, a study conducted in Italy (17) showed that healthcare workers had high-risk perceptions of COVID-19 compared to the general population. It is essential to note that our current study's survey was conducted when Vietnam was still in the unprecedented national lockdown. Therefore, risk perception was likely a key factor for preventing and controlling COVID-19 at the early phase when Vietnam reported no deaths (18).

Our findings also indicate that hospital staff perceived a high risk of transmitting SARS-CoV-2 (score = 3.6). This is consistent with a study conducted in China, which found that most healthcare workers were afraid that they would transmit the virus to others (84.7%) (8). This perception regarding their ability to transmit the virus indicates that hospital staff might often opt to self-isolate or get tested for COVID-19 after their exposure to SARS-CoV-2-positive patients. Moreover, we also found that participants who worked in the Emergency-Intensive Care department were more likely to perceive that they were at a high risk of transmitting the virus, compared to those in Infection Control (Coef. = −0.38) and Preventive Medicine-Public Health-Nutrition departments (Coef. = −0.28). Such a difference may be because staff in the Emergency-Intensive Care department tend to carry out more invasive procedures. Lastly, we observed differences among healthcare workers in their perception regarding the workplace response to COVID-19 (score = 2.0). This perception may have a negative impact on the psychological well-being and performance of healthcare workers as well as an indirect impact on the community. Therefore, it is critical to identify departments with higher perceived and real risks of exposures to ensure adequate provision of PPEs and development of risk communication/management strategies in healthcare setting.

In this study, participants from the central region were less likely to report a perceived exposure to COVID-19 than those from the northern region (OR = 0.52). Population differences may explain this finding. The northern region has ~1,060 people/km^2^ (15). Moreover, hospitals in this region often experience an over-capacity. Our finding is consistent with another study in Vietnam, which links geographical differences to differences in COVID-19 exposure (12). Regarding the perceived exposure risk, nurses reported a lower daily risk of exposure than doctors (51.9 and 53.8%, respectively); however, nurses were also more likely to perceive a risk of exposures to COVID-19 than doctors (OR = 0.56). These findings may be due to the nature of their work in which doctors often perform procedures in closer contact with the patients than nurses, albeit at a lower patient-contact frequency, such as intubation. Therefore, a pandemic support intervention, such as implementation of software to track daily exposure risk, should be prioritized for hospital staff, including doctors, working in population-high density areas.

Evaluating exposure risk perception among healthcare workers plays a crucial role in infectious disease prevention and control effort, especially in the early phase of a pandemic. First, it is urgent to strengthen the provision of PPEs to high-risk departments and of training programs to ensure proper use of PPEs and adherence to other infection control strategies (19) (e.g., hand hygiene and administrative and environmental controls). Together with an enhanced preventive protocols and guidance by the Vietnam Ministry of Health, such interventions should be able to improve the perceived exposure risk of contracting the viruses and reduce their work-related stress (20). Secondly, this study reveals persisting concerns about infections and transmissions among hospital staff. To address this concern, developing a reporting system of health workers' epidemiological histories and contact-tracing systems of patients who have been in contact with healthcare workers for at least 14 days might be worth considering. Besides, the early identification of symptoms and widespread testing for staff also contribute to avoiding nosocomial clusters in hospital (21, 22). Finally, although the present study is conducted within the setting of Vietnam, our findings reveal a high exposure risk perception among healthcare workers that might also exist in other settings. The high perception of exposure risk in this population is an important factor in the overall success of prevention and control efforts against COVID-19.

Our study has limitations. First, the cross-sectional study design limited the identification of a causal link underlying the assessed perception of risk. Second, our data are self-reported, which might suffer from recall bias and social desirability. Finally, the generalizability of our study is limited by the sample size snowball sampling technique.

Despite these limitations, our study provides timely evidence highlighting the high exposure risk perception among an important workforce during a pandemic response to COVID-19. Evaluating exposure risk perception among healthcare workers in the early phase of the pandemic should provide insights to optimize infectious disease prevention and control strategies. First, to address the persisting concern about SARS-CoV-2 infections and transmissions among hospital staff, developing a system to report health workers' COVID-19 epidemiological histories and contact-tracing applications to track COVID-19 patients who have been in contact with healthcare workers might be worth considering. Besides, the early identification of COVID-19 symptoms and enhanced testing services for hospital staff might also contribute to avoiding nosocomial clusters in the hospitals (21, 22). Second, it is urgent to strengthen the provision of PPEs to high-risk departments and of training programs to ensure proper use of PPEs and adherence to other infection control strategies (e.g., hand hygiene and administrative and environmental controls) (19). Together with improved disease prevention protocols and guidance by the Vietnam Ministry of Health, such interventions should be able to improve hospital staff' perceived exposure risk of contracting and transmitting the virus and workplace disease response, thereby potentially reducing their work-related stress and increasing performance (20).

## Conclusion

We identified a persisting gap in the interventions to improve the risk perception toward COVID-19 among hospital staff during the unprecedented lockdown period in Vietnam. A comprehensive approach, including improved risk communications, safety training and psychological support programs for hospital staff, especially those working in high population density areas and in high-risk departments, might further strengthen the national effort to control the pandemic.

## Data Availability Statement

The raw data supporting the conclusions of this article will be made available by the authors, without undue reservation.

## Ethics Statement

The studies involving human participants were reviewed and approved by the Review Committee at Hanoi Medical University on March 27, 2020. The patients/participants provided their written informed consent to participate in this study.

## Author Contributions

XL, QTN, BO, QNN, QP, NT, TN, HL, LV, MH, GV, CL, RH, and CH: conceptualization and writing—review and editing. QP, NT, and TN: data curation. GV and LV: data analysis. XL, QN, QP, NT, and TN: methodology. XL, HL, CL, RH, and CH: supervision. XL, QTN, BO, QNN, and MH: writing-original draft. QP, NT, TN, and MH: project administration. All authors contributed to the article and approved the submitted version.

## Conflict of Interest

The authors declare that the research was conducted in the absence of any commercial or financial relationships that could be construed as a potential conflict of interest.

## Appendix

**Table 5:** Full model.

	**Risk of exposure to COVID-19**		**High-risk individual transmitted COVID-19 from participants**		**Opinion risk of exposure to the COVID-19 from participants**		**Miss conception about exposure risk of COVID-19 from workplace**	
	**OR**	**95% CI**	**Coef**.	**95% CI**	**Coef**.	**95% CI**	**Coef**.	**95% CI**
**Region (vs. Northern)**								
Central	0.53^*^	0.28; 1.03	–0.07	–0.30; 0.16	–0.12	–0.29; 0.06	0.04	–0.10; 0.17
South	0.57	0.24; 1.37	–0.02	–0.36; 0.32	0.09	–0.17; 0.36	–0.01	–0.21; 0.19
Age	1.04	0.98; 1.10	–0.00	–0.02; 0.02	–0.00	–0.02; 0.01	0.01^*^	–0.00; 0.03
**Gender (vs. male)**								
Female	2.22^***^	1.36; 3.64	–0.10	–0.27; 0.08	–0.05	–0.19; 0.09	0.06	–0.04; 0.17
**Living with (vs. family/friends)**								
Alone	0.49	0.19; 1.27	0.20	–0.17; 0.57	0.32^**^	0.03; 0.61	–0.08	–0.30; 0.14
**Marital status (vs. single/separated/widowed)**								
Marriage	0.59	0.30; 1.16	0.35^***^	0.09; 0.61	0.13	–0.07; 0.33	–0.21^***^	–0.36; –0.06
**Education (vs. university and below)**								
Postgraduate degree	0.79	0.45; 1.38	–0.04	–0.25; 0.17	–0.00	–0.17; 0.16	–0.10	–0.22; 0.03
**Occupation (vs. doctor)**								
Nurse	0.45^**^	0.23; 0.87	0.20	–0.04; 0.44	0.01	–0.17; 0.20	–0.20^***^	–0.34; –0.06
Others	0.88	0.49; 1.59	0.11	–0.12; 0.33	–0.11	–0.28; 0.07	0.13^*^	–0.01; 0.26
**Level of hospital (vs. central level)**								
Provincial level	0.86	0.46; 1.61	0.08	–0.16; 0.32	–0.03	–0.22; 0.15	–0.06	–0.20; 0.08
District health center	0.73	0.35; 1.52	–0.00	–0.28; 0.28	0.04	–0.18; 0.26	–0.12	–0.29; 0.04
Others	0.91	0.51; 1.64	–0.01	–0.24; 0.21	0.00	–0.17; 0.18	0.07	–0.06; 0.20
**Department (vs. emergency-intensive care)**								
Internal medicine	0.40^*^	0.15; 1.09	0.18	–0.19; 0.54	0.11	–0.17; 0.40	0.05	–0.17; 0.27
Surgery-obstetrics-pediatrics	0.59	0.23; 1.49	0.05	–0.32; 0.42	–0.03	–0.32; 0.26	0.11	–0.11; 0.33
Imaging diagnosis-scientific laboratory—clinic	0.27^***^	0.10; 0.70	0.00	–0.35; 0.36	–0.05	–0.33; 0.22	–0.05	–0.26; 0.16
Administrative offices	0.70	0.27; 1.79	–0.22	–0.60; 0.15	–0.28^*^	–0.57; 0.01	–0.01	–0.23; 0.22
Infectious disease-infection control	0.41	0.12; 1.39	–0.38^*^	–0.83; 0.07	–0.30^*^	–0.64; 0.05	0.06	–0.21; 0.32
Preventive medicine-public health-nutrition	0.61	0.23; 1.60	–0.26	–0.65; 0.12	–0.26^*^	–0.55; 0.04	0.00	–0.22; 0.23
Others	0.38^**^	0.15; 0.94	–0.08	–0.42; 0.27	–0.21	–0.48; 0.05	0.16	–0.05; 0.36
Years of career (years)	0.95^*^	0.90; 1.00	–0.01	–0.03; 0.01	0.01	–0.01; 0.02	–0.01	–0.02; 0.00
Number of diagnosed COVID-19 patients to whom you believed to be exposed	0.85	0.65; 1.13	0.00	–0.02; 0.02	0.01	–0.00; 0.02	–0.00	–0.01; 0.01
Number of undiagnosed COVID-19 patients to whom you believed to be exposed	0.98	0.95; 1.01	–0.00	–0.00; 0.00	0.00	–0.00; 0.00	0.00	–0.00; 0.00

*** *p < 0.01*,

** *p < 0.05*,

* *p < 0.1*.
